# Supplementary material for: Impact of fluoroquinolone resistance on the cost-effectiveness of empiric treatment for multidrug- or rifampicin-resistant tuberculosis
Source: PLOS Glob Public Health. 2025 Oct 16;5(10):e0005275. doi: 10.1371/journal.pgph.0005275 (PMC12530546; doi:10.1371/journal.pgph.0005275)
Supplement: S1 Text — (DOCX) [file pgph.0005275.s002.docx]

**S1 Text. Supplementary methods**

*Model Structure and Assumptions*

The model incorporated monthly transitions between states, including ongoing treatment, loss to follow-up, treatment failure, or death consistent with the previous model [(1)](https://sciwheel.com/work/citation?ids=15750681&pre=&suf=&sa=0). The treatment duration was set at 24 weeks in line with the TB-PRACTECAL protocol [(2)](https://sciwheel.com/work/citation?ids=13182382&pre=&suf=&sa=0). Following the previous model structure, treatment failures transitioned to either a short (36-week) or long (80-week) traditional 'rescue' regimen based on country-specific proportional standards. Patients who failed this ‘rescue’ regimen progressed to death. Patients lost to follow-up could re-enter treatment within two years, while those successfully treated faced potential relapse within four years of completion. Patients experiencing relapse were eligible for re-entry to treatment, and the transition rate back into this state was parameterised based on national TB treatment coverage rates.

As in the previous model, we maintained a conservative analytical approach, assuming no significant impact on mortality, treatment failure or disease recurrence and attributing improvements in outcomes primarily to reductions in loss to follow-up. We did not include a comparison with the BPaLC regimen, as the WHO Guideline Development Group recommended against its use due to the increased pill burden and adverse effects associated with clofazimine use with limited benefit [(3)](https://sciwheel.com/work/citation?ids=15669401&pre=&suf=&sa=0).

*Extended Sensitivity Analyses*

We assessed the impact of varying an extended set of parameters by setting each parameter to predefined low and high values (as specified in **S3 Table**) while keeping all other variables fixed at their baseline values. These extended parameters included fluoroquinolone resistance prevalence, the difference in risk ratios between BPaLM and BPaL compared to traditional regimens, moxifloxacin and bedaquiline prices, discount rates for costs and outcomes, rates of return from loss to follow-up, risk of relapse, non-drug costs, and adverse event costs. To visualise these results, we created tornado diagrams (**S4 Fig**) to rank parameters by their relative impact on ICER. In addition, we used cost-effectiveness planes (**S5 Fig**) to illustrate incremental costs and DALYs averted across the parameter ranges.

[*Bibliography*](https://sciwheel.com/work/bibliography)

[1. Sweeney S, Berry C, Kazounis E, Motta I, Vassall A, Dodd M, et al. Cost-effectiveness of short, oral treatment regimens for rifampicin resistant tuberculosis. PLOS Glob Public Health. 2022 Dec 7;2(12):e0001337.](https://sciwheel.com/work/bibliography/15750681)

[2. Berry C, du Cros P, Fielding K, Gajewski S, Kazounis E, McHugh TD, et al. TB-PRACTECAL: study protocol for a randomised, controlled, open-label, phase II-III trial to evaluate the safety and efficacy of regimens containing bedaquiline and pretomanid for the treatment of adult patients with pulmonary multidrug-resistant tuberculosis. Trials. 2022 Jun 13;23(1):484.](https://sciwheel.com/work/bibliography/13182382)

[3. Vanino E, Granozzi B, Akkerman OW, Munoz-Torrico M, Palmieri F, Seaworth B, et al. Update of drug-resistant tuberculosis treatment guidelines: A turning point. Int J Infect Dis. 2023 May;130 Suppl 1:S12–5.](https://sciwheel.com/work/bibliography/15669401)
