# Supplementary material for: Impact of fluoroquinolone resistance on the cost-effectiveness of empiric treatment for multidrug- or rifampicin-resistant tuberculosis
Source: PLOS Glob Public Health. 2025 Oct 16;5(10):e0005275. doi: 10.1371/journal.pgph.0005275 (PMC12530546; doi:10.1371/journal.pgph.0005275)
Supplement: S2 Text — (DOCX) [file pgph.0005275.s003.docx]

**S2 Text. Updated efficacy model.** Instead of combining outcomes for patients with MDR/RR-TB and pre-XDR-TB into a single cohort, we stratified these populations into two distinct groups. The first group consisted of patients with true MDR/RR-TB susceptible to fluoroquinolones, while the second group consisted of patients with pre-XDR-TB. This stratification allowed us to model empirical treatment scenarios according to resistance patterns, using different efficacies for MDR/RR-TB and pre-XDR-TB. MDR/RR-TB multidrug- or rifampicin-resistant tuberculosis; pre-XDR-TB pre-extensively drug-resistant TB.


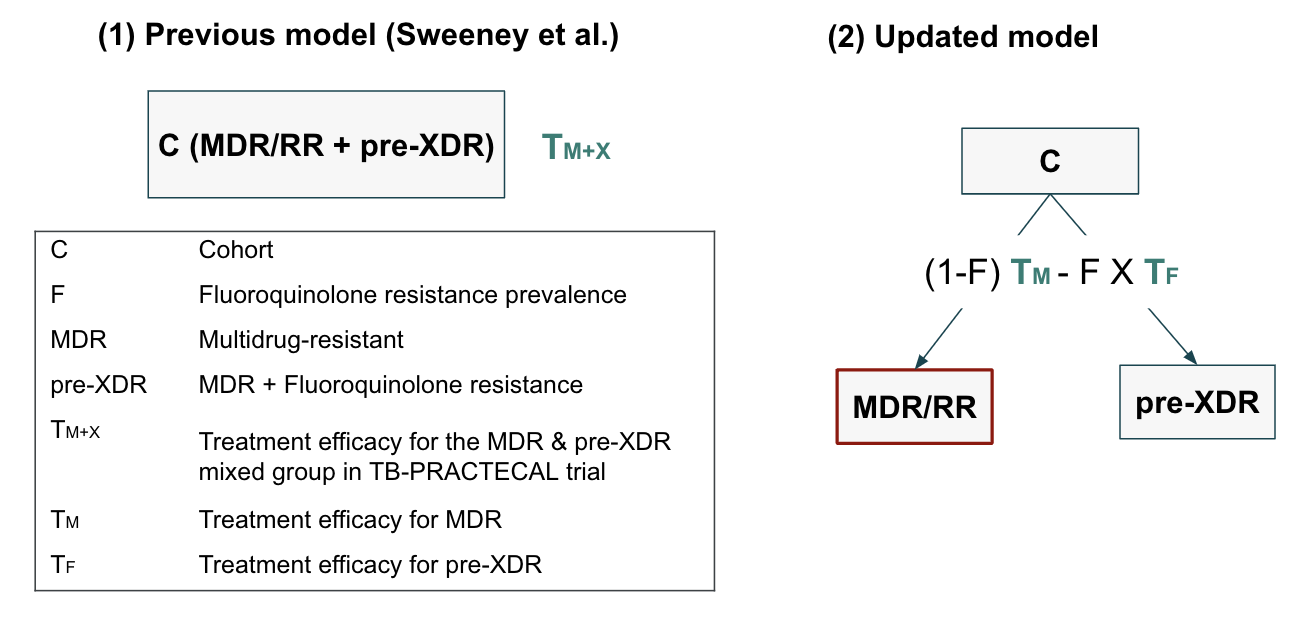
​
