# Supplementary material for: Impact of fluoroquinolone resistance on the cost-effectiveness of empiric treatment for multidrug- or rifampicin-resistant tuberculosis
Source: PLOS Glob Public Health. 2025 Oct 16;5(10):e0005275. doi: 10.1371/journal.pgph.0005275 (PMC12530546; doi:10.1371/journal.pgph.0005275)

**S1 Table. Treatment scenarios.** The table shows two empirical treatment scenarios (BPaL and BPaLM) for patients not tested for fluoroquinolone resistance. Each row represents a treatment option, and columns show outcomes for true MDR/RR-TB and pre-XDR-TB patients.


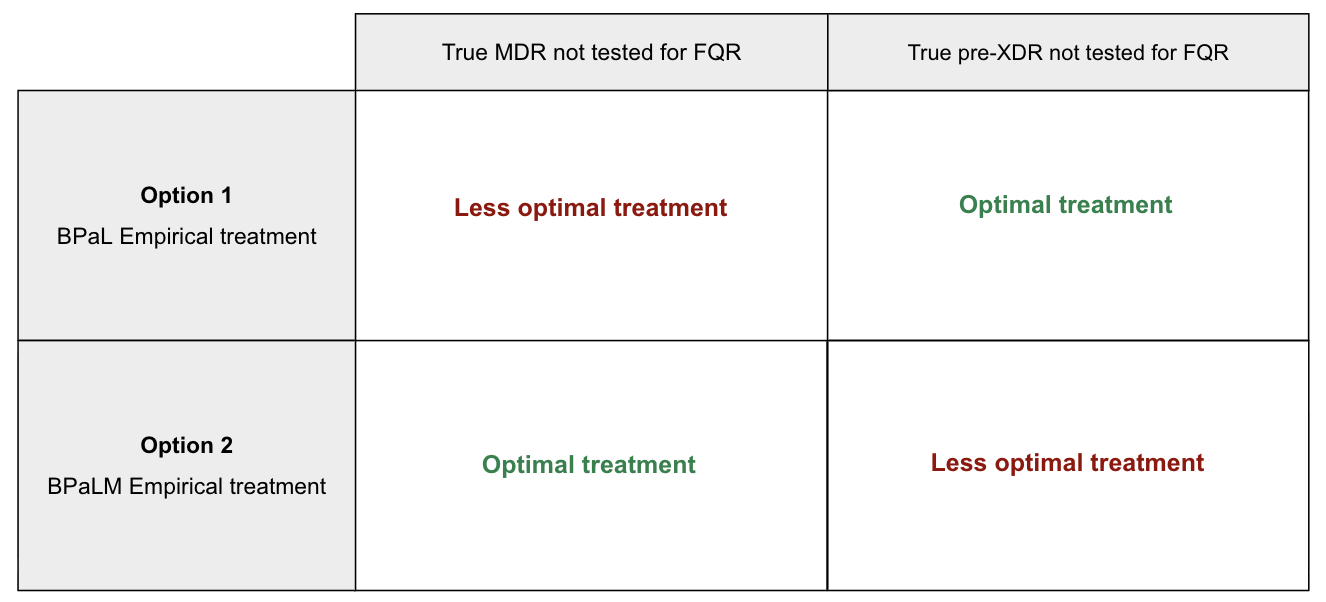

Supplement: S1 Table — BPaL bedaquiline, pretomanid and linezolid; BPaLM bedaquiline, pretomanid, linezolid and moxifloxacin; MDR/RR-TB multidrug- or rifampicin-resistant tuberculosis; pre-XDR-TB pre-extensively drug-resistant TB. (DOCX) [file pgph.0005275.s004.docx]
