# Supplementary material for: Impact of fluoroquinolone resistance on the cost-effectiveness of empiric treatment for multidrug- or rifampicin-resistant tuberculosis
Source: PLOS Glob Public Health. 2025 Oct 16;5(10):e0005275. doi: 10.1371/journal.pgph.0005275 (PMC12530546; doi:10.1371/journal.pgph.0005275)
Supplement: S2 Table — (DOCX) [file pgph.0005275.s005.docx]

**S2 Table. Country-specific and non-specific parameters.** As in Sweeney et al. [(1)](https://sciwheel.com/work/citation?ids=15750681&pre=&suf=&sa=0), we used a provider perspective for cost analysis, using the Value TB dataset, published literature and expert opinion. The tables include (1) country-specific drug regimens for BPaL, BPaLM and standard care, (2) model parameters and DALY weights for all health states in the model, and (3) unit costs. Both (2) and (3) provide baseline values as well as standard errors and PSA distributions for probabilistic sensitivity analysis. Analyses were conducted using 2019 USD as the reference currency, with a standard annual discount rate of 3% applied to both costs and DALYs. All values specified in (1), (2) and (3) were derived from Sweeney et al. [(1)](https://sciwheel.com/work/citation?ids=15750681&pre=&suf=&sa=0). BPaL: bedaquiline, pretomanid and linezolid; BPaLM: bedaquiline, pretomanid, linezolid and moxifloxacin; DALY: disability-adjusted life year; PSA: probabilistic sensitivity analysis; USD: United States dollars.

1. **Regimen specification**

| **Regimen** | **Georgia** | **India** | **Philippines** | **South Africa** |
| --- | --- | --- | --- | --- |
| BPaL regimen | Bdq, Pa, L (400 mg once daily for 2 weeks followed by 200 mg 3 times per week for 22 weeks 200mg once daily 600mg daily for 16 weeks then 300mg daily for the remaining 8 weeks or earlier when moderately tolerated) | | | |
| BPaLM regimen | Bdq, Pa, L, Mfx (400 mg once daily for 2 weeks followed by 200 mg 3 times per week for 22 weeks 200mg once daily 600mg daily for 16 weeks then 300mg daily for the remaining 8 weeks or earlier when moderately tolerated 400 mg once daily) | | | |
| Primary short traditional regimen* | Am, Mfx, Cfz, Z, E, Hh, Pto; | Lfx, Cfz, Z, E, Hh, Eto, Bdq; | Lfx, Cfz, Z, E, Pto, Hh, Bdq; | Lfx, Lzd, Cfz, Z, E, Hh, Bdq; |
|  | Mfx, Cfz, Z, E | Lfx, Cfz, Z, E | Lfx, Cfz, Z, E | Lfx, Lzd, Cfz, Z, E |
| Primary long traditional regimen* | Lfx, Lzd, Cfz, Bdq | Lfx, Lzd, Cfz, Cs, Bdq | Lfx, Lzd, Cfz, Bdq | Lfx, Trd, Lzd, Cfz, Bdq |
| Percent MDR/RR-TB patients on short /  long traditional regimens (traditional mix)* | 31% / 69% | 96% / 4% | 99% / 1% | 74% / 26% |

Am amikacin; Bdq bedaquiline; BPaL bedaquiline, pretomanid and linezolid; BPaLM bedaquiline, pretomanid, linezolid and moxifloxacin; Cfz clofazimine; E ethambutol; Eto ethionamide; Hh high-dose isoniazid; Lfx levofloxacin; L Linezolid; Mfx Moxifloxacin; Pa Pretomanid; Pto prothionamide; Trd terizidone; Z pyrazinamide.

* These treatment regimens and the distribution of patients between short and long regimens are documented in Sweeney et al. [(1)](https://sciwheel.com/work/citation?ids=15750681&pre=&suf=&sa=0) using 2019 data.

1. **Model parameters and DALY weights for all health states in the model**

| **Parameter or condition** | **Baseline Value** | **SE** | **PSA Distribution** | **Health state description (DALYs)** | **Reference** |
| --- | --- | --- | --- | --- | --- |
| Model parameters | | | | | |
| Average age at model start (years) | 35 |  | N/A |  | [(1)](https://sciwheel.com/work/citation?ids=15750681&pre=&suf=&sa=0) |
| Average body weight (kilograms) | 51-70 |  | N/A |  | [(1)](https://sciwheel.com/work/citation?ids=15750681&pre=&suf=&sa=0) |
| Discount rates for costs and effects | 3% |  | N/A |  | [(1)](https://sciwheel.com/work/citation?ids=15750681&pre=&suf=&sa=0) |
| Annual risk of relapse in year 1 post-treatment | 2.80% | 0.40% | Normal |  | [(1)](https://sciwheel.com/work/citation?ids=15750681&pre=&suf=&sa=0) |
| Annual risk of relapse in year 2 post-treatment | 1.00% | 0.30% | Normal |  | [(1)](https://sciwheel.com/work/citation?ids=15750681&pre=&suf=&sa=0) |
| Annual risk of relapse in year 3 post-treatment | 0.40% | 0.20% | Normal |  | [(1)](https://sciwheel.com/work/citation?ids=15750681&pre=&suf=&sa=0) |
| Annual risk of relapse in year 4 post-treatment | 0.30% | 0.20% | Normal |  | [(1)](https://sciwheel.com/work/citation?ids=15750681&pre=&suf=&sa=0) |
| Hazard ratio for relapse among people with HIV | 2.40 |  | N/A |  | [(1)](https://sciwheel.com/work/citation?ids=15750681&pre=&suf=&sa=0) |
| Annual likelihood of return to care after LTFU | 28% | 3% | Normal |  | [(1)](https://sciwheel.com/work/citation?ids=15750681&pre=&suf=&sa=0) |
| Access to end of life care | 25% | 5% | Normal |  | [(1)](https://sciwheel.com/work/citation?ids=15750681&pre=&suf=&sa=0) |
| Monthly probability of death for end-of-life care | 6.86% | 0.69% | Beta |  | [(1)](https://sciwheel.com/work/citation?ids=15750681&pre=&suf=&sa=0) |
| Monthly probability of death following LTFU | 6.86% | 0.69% | Beta |  | [(1)](https://sciwheel.com/work/citation?ids=15750681&pre=&suf=&sa=0) |
| DALY weights for all health states in the model | | | | | |
| Post-TB | 0.053 |  | Beta |  | [(1)](https://sciwheel.com/work/citation?ids=15750681&pre=&suf=&sa=0) |
| HIV, no active TB | 0.125 | 0.07 | Beta | Has weight loss, fatigue, and frequent infections. | [(1)](https://sciwheel.com/work/citation?ids=15750681&pre=&suf=&sa=0) |
| Active TB, no HIV | 0.333 | 0.06 | Beta | Has a persistent cough and fever, is short of breath, feels weak, and has lost a lot of weight. | [(1)](https://sciwheel.com/work/citation?ids=15750681&pre=&suf=&sa=0) |
| Active TB & HIV | 0.439 | 0.02 | Beta | combined disability weight | [(1)](https://sciwheel.com/work/citation?ids=15750681&pre=&suf=&sa=0) |
| End of life | 0.540 | 0.09 | Beta | Has lost a lot of weight and regularly uses strong medication to avoid constant pain. The person has no appetite, feels nauseous, and needs to spend most of the day in bed. | [(1)](https://sciwheel.com/work/citation?ids=15750681&pre=&suf=&sa=0) |
| Death | 1 |  | Beta |  | [(1)](https://sciwheel.com/work/citation?ids=15750681&pre=&suf=&sa=0) |
| Liver dysfunction (grade 3 and above) | 0 | - | Beta | Asymptomatic | [(1)](https://sciwheel.com/work/citation?ids=15750681&pre=&suf=&sa=0) |
| Pancreatitis (grade 3 and above) | 0.114 | 0.02 | Beta | Has pain in the belly and feels nauseous. The person has difficulties with daily activities. | [(1)](https://sciwheel.com/work/citation?ids=15750681&pre=&suf=&sa=0) |
| Anaemia (grade 3 and above) | 0.052 | 0.01 | Beta | Has moderate fatigue, weakness, and shortness of breath after exercise, making daily activities more difficult. | [(1)](https://sciwheel.com/work/citation?ids=15750681&pre=&suf=&sa=0) |
| Neutropenia (grade 3 and above) | 0 | - | Beta | Asymptomatic | [(1)](https://sciwheel.com/work/citation?ids=15750681&pre=&suf=&sa=0) |
| QTcF prolongation (grade 3 or above) | 0 | - | Beta | Asymptomatic | [(1)](https://sciwheel.com/work/citation?ids=15750681&pre=&suf=&sa=0) |
| Vomiting (grade 3 and above) | 0.114 | 0.02 | Beta | Has pain in the belly and feels nauseous. The person has difficulties with daily activities. | [(1)](https://sciwheel.com/work/citation?ids=15750681&pre=&suf=&sa=0) |
| Renal dysfunction (grade 3 and above) | 0.051 | 0.01 | Beta | Has fever and aches, and feels weak, which causes some difficulty with daily activities. | [(1)](https://sciwheel.com/work/citation?ids=15750681&pre=&suf=&sa=0) |

DALY disability-adjusted life year; HIV human immunodeficiency virus; LTFU loss to follow-up; N/A not applicable; PSA probabilistic sensitivity analysis**;** QT corrected for heart rate by Fridericia's cube root formula; SE standard error.

1. **Unit costs**

| **Service** | **Georgia*** | **India*** | **South Africa*** | **Philippines*** | **PSA**  **Distribution** | **Source** |
| --- | --- | --- | --- | --- | --- | --- |
| Outpatient diagnostic visit | $3.69 ($0.70) | $1.11 ($0.21) | $12.00 ($2.26) | $1.99 ($0.41) | Gamma | [(1)](https://sciwheel.com/work/citation?ids=15750681&pre=&suf=&sa=0) |
| Outpatient treatment support visit | $2.42 ($0.45) | $1.44 ($0.27) | $12.00 ($2.26) | $0.42 ($0.08) | Gamma | [(1)](https://sciwheel.com/work/citation?ids=15750681&pre=&suf=&sa=0) |
| Outpatient treatment visit | $2.13 ($0.33) | $1.15 ($0.22) | $12.00 ($2.26) | $1.68 ($0.29) | Gamma | [(1)](https://sciwheel.com/work/citation?ids=15750681&pre=&suf=&sa=0) |
| Outpatient monitoring visit | $3.59 ($0.65) | $2.81 ($0.53) |  | $2.05 ($0.36) | Gamma | [(1)](https://sciwheel.com/work/citation?ids=15750681&pre=&suf=&sa=0) |
| Inpatient bed-day | $38.28 ($3.09) | $18.06 ($3.41) | $49.03 ($3.96) | $26.63 ($2.03) | Gamma | [(1)](https://sciwheel.com/work/citation?ids=15750681&pre=&suf=&sa=0) |
| Community-level treatment visit | $1.88 ($0.12) | $0.54 ($0.10) |  |  | Gamma | [(1)](https://sciwheel.com/work/citation?ids=15750681&pre=&suf=&sa=0) |
| Community-level other visit |  | $1.70 ($0.32) |  |  | Gamma | [(1)](https://sciwheel.com/work/citation?ids=15750681&pre=&suf=&sa=0) |
| Lost to follow-up tracing: phone calls | $1.24 ($0.22) |  |  | $0.70 ($0.12) | Gamma | [(1)](https://sciwheel.com/work/citation?ids=15750681&pre=&suf=&sa=0) |
| Lost to follow-up tracing: home visit |  |  | $9.43 ($2.57) |  | Gamma | [(1)](https://sciwheel.com/work/citation?ids=15750681&pre=&suf=&sa=0) |
| Phone consultation | $0.53 ($0.10) |  |  |  | Gamma | [(1)](https://sciwheel.com/work/citation?ids=15750681&pre=&suf=&sa=0) |
| Contact tracing | $0.50 ($0.30) |  |  |  | Gamma | [(1)](https://sciwheel.com/work/citation?ids=15750681&pre=&suf=&sa=0) |
| Lab tests |  |  |  |  |  | [(1)](https://sciwheel.com/work/citation?ids=15750681&pre=&suf=&sa=0) |
| Sputum collection | $1.63 ($0.24) |  |  |  | Gamma | [(1)](https://sciwheel.com/work/citation?ids=15750681&pre=&suf=&sa=0) |
| Ziehl-Neelsen smear microscopy | $5.59 ($0.84) | $1.90 ($0.36) | $6.95 ($1.04) | $2.53 ($0.39) | Gamma | [(1)](https://sciwheel.com/work/citation?ids=15750681&pre=&suf=&sa=0) |
| Solid sputum culture | $8.56 ($1.29) | $1.90 ($0.36) |  | $23.29 ($3.17) | Gamma | [(1)](https://sciwheel.com/work/citation?ids=15750681&pre=&suf=&sa=0) |
| Sputum culture | $15.55 ($2.33) |  |  |  | Gamma | [(1)](https://sciwheel.com/work/citation?ids=15750681&pre=&suf=&sa=0) |
| Electrocardiogram | $1.89 ($0.26) | $0.99 ($0.19) | $14.35 ($2.15) | $3.50 ($0.63) | Gamma | [(1)](https://sciwheel.com/work/citation?ids=15750681&pre=&suf=&sa=0) |
| HIV rapid test | $2.58 ($0.28) | $1.29 ($0.24) |  | $3.11 ($0.42) | Gamma | [(1)](https://sciwheel.com/work/citation?ids=15750681&pre=&suf=&sa=0) |
| Full haemogram | $4.01 ($0.50) | $0.65 ($0.12) |  | $2.69 ($0.96) | Gamma | [(1)](https://sciwheel.com/work/citation?ids=15750681&pre=&suf=&sa=0) |
| Creatinine |  | $0.59 ($0.11) |  | $1.78 ($0.27) | Gamma | [(1)](https://sciwheel.com/work/citation?ids=15750681&pre=&suf=&sa=0) |
| Blood sugar | $1.01 ($0.15) | $0.78 ($0.15) |  | $2.75 ($0.59) | Gamma | [(1)](https://sciwheel.com/work/citation?ids=15750681&pre=&suf=&sa=0) |
| Thyroid-stimulating hormone test |  | $2.69 ($0.51) |  |  | Gamma | [(1)](https://sciwheel.com/work/citation?ids=15750681&pre=&suf=&sa=0) |
| Chest Xray (film) | $4.45 ($0.44) | $1.99 ($0.37) |  | $2.75 ($0.51) | Gamma | [(1)](https://sciwheel.com/work/citation?ids=15750681&pre=&suf=&sa=0) |
| Chest Xray (digital) | $2.63 ($0.84) | $2.05 ($0.39) |  | $1.97 ($0.50) | Gamma | [(1)](https://sciwheel.com/work/citation?ids=15750681&pre=&suf=&sa=0) |
| Fasting blood sugar |  |  |  | $1.55 ($0.23) | Gamma | [(1)](https://sciwheel.com/work/citation?ids=15750681&pre=&suf=&sa=0) |
| Liver function test | $2.54 ($0.38) | $2.69 ($0.51) |  | $2.92 ($0.33) | Gamma | [(1)](https://sciwheel.com/work/citation?ids=15750681&pre=&suf=&sa=0) |
| Audiometry |  |  |  | $8.80 ($2.33) | Gamma | [(1)](https://sciwheel.com/work/citation?ids=15750681&pre=&suf=&sa=0) |
| Visual acuity |  |  |  | $7.54 ($1.92) | Gamma | [(1)](https://sciwheel.com/work/citation?ids=15750681&pre=&suf=&sa=0) |
| Potassium |  |  |  | $5.37 ($0.81) | Gamma | [(1)](https://sciwheel.com/work/citation?ids=15750681&pre=&suf=&sa=0) |
| Biochemistry | $1.79 ($0.24) |  | $32.30 ($4.85) | $2.46 ($1.27) | Gamma | [(1)](https://sciwheel.com/work/citation?ids=15750681&pre=&suf=&sa=0) |
| Electrolyte test |  |  |  | $0.95 ($0.14) | Gamma | [(1)](https://sciwheel.com/work/citation?ids=15750681&pre=&suf=&sa=0) |
| Bloodgroup RH | $2.54 ($0.38) |  |  | $1.74 ($0.26) | Gamma | [(1)](https://sciwheel.com/work/citation?ids=15750681&pre=&suf=&sa=0) |
| Blood clotting | $6.55 ($1.24) |  |  |  | Gamma | [(1)](https://sciwheel.com/work/citation?ids=15750681&pre=&suf=&sa=0) |
| Light-emitting diode fluorescence microscopy (LED-FM) | $2.52 ($0.38) |  |  |  | Gamma | [(1)](https://sciwheel.com/work/citation?ids=15750681&pre=&suf=&sa=0) |
| Magnetic resonance imaging | $2.26 ($0.34) |  |  |  | Gamma | [(1)](https://sciwheel.com/work/citation?ids=15750681&pre=&suf=&sa=0) |
| Computerized Tomography (CT) scan | $10.10 ($1.52) |  |  |  | Gamma | [(1)](https://sciwheel.com/work/citation?ids=15750681&pre=&suf=&sa=0) |
| Ultrasound test |  | $0.89 ($0.17) | $6.95 ($1.04) |  | Gamma | [(1)](https://sciwheel.com/work/citation?ids=15750681&pre=&suf=&sa=0) |
| Other tests | $3.55 ($0.53) |  |  |  |  |  |
| Cost per month for antiretroviral therapy | $23.48 ($3.52) |  |  |  | Gamma | [(1)](https://sciwheel.com/work/citation?ids=15750681&pre=&suf=&sa=0) |
| Cost per month for LTFU patients | $3.13 ($0.76) |  |  |  | Gamma | [(1)](https://sciwheel.com/work/citation?ids=15750681&pre=&suf=&sa=0) |
| Cost per month for end of life state | $3.69 ($0.70) | $1.11 ($0.21) | $12.00 ($2.26) | $1.99 ($0.41) | Gamma | [(1)](https://sciwheel.com/work/citation?ids=15750681&pre=&suf=&sa=0) |
| Cost per death | $2.42 ($0.45) | $1.44 ($0.27) | $12.00 ($2.26) | $0.42 ($0.08) | n/a | [(1)](https://sciwheel.com/work/citation?ids=15750681&pre=&suf=&sa=0) |
| Cost after TB cure | $2.13 ($0.33) | $1.15 ($0.22) | $12.00 ($2.26) | $1.68 ($0.29) | n/a | [(1)](https://sciwheel.com/work/citation?ids=15750681&pre=&suf=&sa=0) |
| Cost per month for liver dysfunction | $3.59 ($0.65) | $2.81 ($0.53) |  | $2.05 ($0.36) | Gamma | [(1)](https://sciwheel.com/work/citation?ids=15750681&pre=&suf=&sa=0) |
| Cost per month for pancreatitis | $38.28 ($3.09) | $18.06 ($3.41) | $49.03 ($3.96) | $26.63 ($2.03) | Gamma | [(1)](https://sciwheel.com/work/citation?ids=15750681&pre=&suf=&sa=0) |
| Cost per month for anaemia | $1.88 ($0.12) | $0.54 ($0.10) |  |  | Gamma | [(1)](https://sciwheel.com/work/citation?ids=15750681&pre=&suf=&sa=0) |
| Cost per month for neutropenia |  | $1.70 ($0.32) |  |  | Gamma | [(1)](https://sciwheel.com/work/citation?ids=15750681&pre=&suf=&sa=0) |
| Cost per month for QTcF prolongation | $1.24 ($0.22) |  |  | $0.70 ($0.12) | Gamma | [(1)](https://sciwheel.com/work/citation?ids=15750681&pre=&suf=&sa=0) |
| Cost per month for vomiting |  |  | $9.43 ($2.57) |  | Gamma | [(1)](https://sciwheel.com/work/citation?ids=15750681&pre=&suf=&sa=0) |
| Cost per month for renal dysfunction | $0.53 ($0.10) |  |  |  | Gamma | [(1)](https://sciwheel.com/work/citation?ids=15750681&pre=&suf=&sa=0) |

* Values presented as: Baseline value (standard error)

CT: computerised tomography; HIV: human immunodeficiency virus; LED-FM: light-emitting diode fluorescence microscopy; LTFU: loss to follow-up; MRI: magnetic resonance imaging; n/a: not applicable; PSA: probabilistic sensitivity analysis; QTcF: QT interval corrected for heart rate using Fridericia's formula; RH: rhesus factor; TB: tuberculosis; USD: United States dollars.
