# Supplementary material for: Impact of fluoroquinolone resistance on the cost-effectiveness of empiric treatment for multidrug- or rifampicin-resistant tuberculosis
Source: PLOS Glob Public Health. 2025 Oct 16;5(10):e0005275. doi: 10.1371/journal.pgph.0005275 (PMC12530546; doi:10.1371/journal.pgph.0005275)
Supplement: S3 Table — (DOCX) [file pgph.0005275.s006.docx]

**S3 Table. Parameter ranges used in univariate sensitivity analyses.** The table shows the parameter ranges used for univariate sensitivity analyses to explore their impact on the ICER. Parameters include fluoroquinolone resistance prevalence (halved and doubled from baseline), difference in risk ratios (RR) between BPaLM and BPaL compared to traditional regimens, moxifloxacin and bedaquiline prices (halved and doubled), discount rates for costs and outcomes (0%, 3% and 6%), rates of return from loss to follow-up, risk of relapse, non-drug costs, and adverse event costs (all halved and doubled). These varied values (low and high) differ from those used for the PSA in **S2 Table (2) and (3)**, as the PSA captures parameter uncertainty based on data, whereas the univariate analysis tests the robustness of conclusions across plausible scenarios. BPaL: bedaquiline, pretomanid and linezolid; BPaLM: bedaquiline, pretomanid, linezolid and moxifloxacin; FQ: fluoroquinolone; ICER: incremental cost-effectiveness ratio; RR: risk ratio.

| **Parameter*** | **Low** | **Base** | **High** |
| --- | --- | --- | --- |
| FQ resistance prevalence | Baseline FQR** × 1/2 | Baseline FQR** | Baseline FQR** × 2 |
| ΔRR between BPaLM and BPaL | 0.06 | 0.12 | 0.18 |
| Moxifloxacin price | 0.8 | 0.16 | 0.32 |
| Bedaquline price | 0.905 | 1.81 | 3.62 |
| Discount rate for costs | 0% | 3.0% | 6.0% |
| Discount rate for outcomes | 0% | 3.0% | 6.0% |
| Rate of return from loss to follow-up | 14% | 28% | 56% |
| Risk of relapse | 0.5 | 1 | 2 |
| Non-drug costs | 0.5 | 1 | 2 |
| Adverse event costs | 0.5 | 1 | 2 |

* All costs are in USD

** Baseline FQR: Georgia 27%; India 37%; Philippines 9%; South Africa 19%.
