# Supplementary material for: Impact of fluoroquinolone resistance on the cost-effectiveness of empiric treatment for multidrug- or rifampicin-resistant tuberculosis
Source: PLOS Glob Public Health. 2025 Oct 16;5(10):e0005275. doi: 10.1371/journal.pgph.0005275 (PMC12530546; doi:10.1371/journal.pgph.0005275)
Supplement: S4 Table — (DOCX) [file pgph.0005275.s007.docx]

**S4 Table. Breakdown of per-person costs by treatment regimen and country.** Cost components per person for BPaL and BPaLM regimens across four countries (Georgia, India, Philippines, and South Africa), including HIV treatment, intervention-related costs (visits and drugs), rescue treatment, management costs (loss to follow-up and adverse events), and end-stage costs (end-of-life care and death). BPaL: bedaquiline, pretomanid, and linezolid; BPaLM: bedaquiline, pretomanid, linezolid, and moxifloxacin.

| **Country** | **Regimen** | **HIV treatment costs*** | **Intervention visit costs*** | **Intervention drug costs*** | **Rescue treatment costs*** | **Loss to follow-up costs*** | **Adverse event costs*** | **End-of-life care costs*** | **Death costs*** | **Total discounted costs*** |
| --- | --- | --- | --- | --- | --- | --- | --- | --- | --- | --- |
| **Georgia** | **BPaL** | 64 | 2,301 | 572 | 250 | 9 | 12 | 12 | 15 | 3,236 |
|  | **BPaLM** | 71 | 2,333 | 602 | 260 | 3 | 18 | 14 | 12 | 3,313 |
| **India** | **BPaL** | 35 | 204 | 532 | 40 | 14 | 9 | 11 | 25 | 869 |
|  | **BPaLM** | 38 | 206 | 557 | 41 | 10 | 14 | 11 | 22 | 899 |
| **Philippines** | **BPaL** | 22 | 418 | 567 | 32 | 10 | 14 | 1 | 20 | 1,083 |
|  | **BPaLM** | 25 | 427 | 599 | 37 | 6 | 22 | 1 | 16 | 1,132 |
| **South Africa** | **BPaL** | 1,379 | 1,236 | 554 | 74 | 72 | 36 | 2 | 26 | 3,380 |
|  | **BPaLM** | 1,527 | 1,257 | 583 | 82 | 35 | 54 | 2 | 23 | 3,564 |

*All costs are presented per person in 2019 US dollars ($2019)
