# Supplementary figures and images for: Impact of fluoroquinolone resistance on the cost-effectiveness of empiric treatment for multidrug- or rifampicin-resistant tuberculosis
Source: PLOS Glob Public Health. 2025 Oct 16;5(10):e0005275. doi: 10.1371/journal.pgph.0005275 (PMC12530546; doi:10.1371/journal.pgph.0005275)

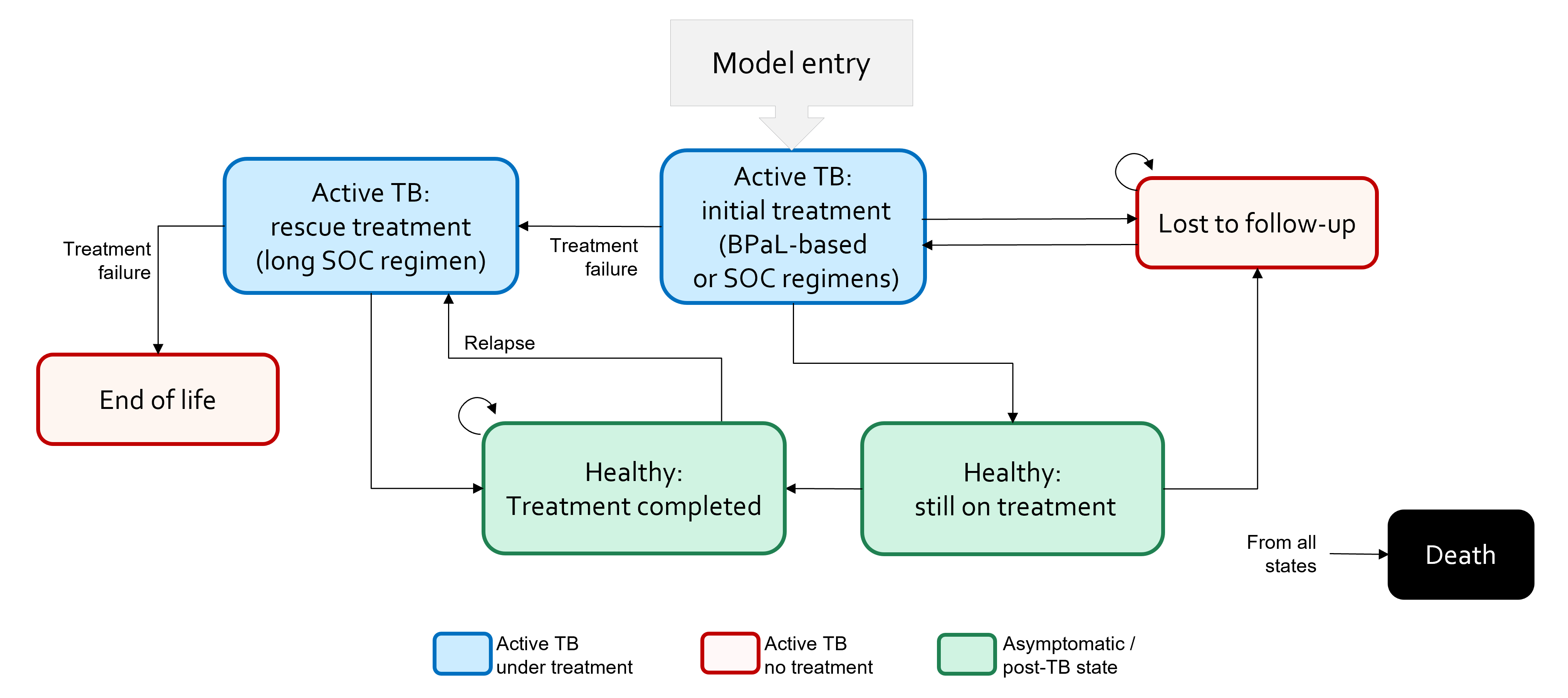

Supplement: S1 Fig — The model structure is based on Sweeney et al. [12]. BPaL, bedaquiline, pretomanid, and linezolid; SOC, standard of care; TB, tuberculosis. (TIF) [file pgph.0005275.s008.tif]

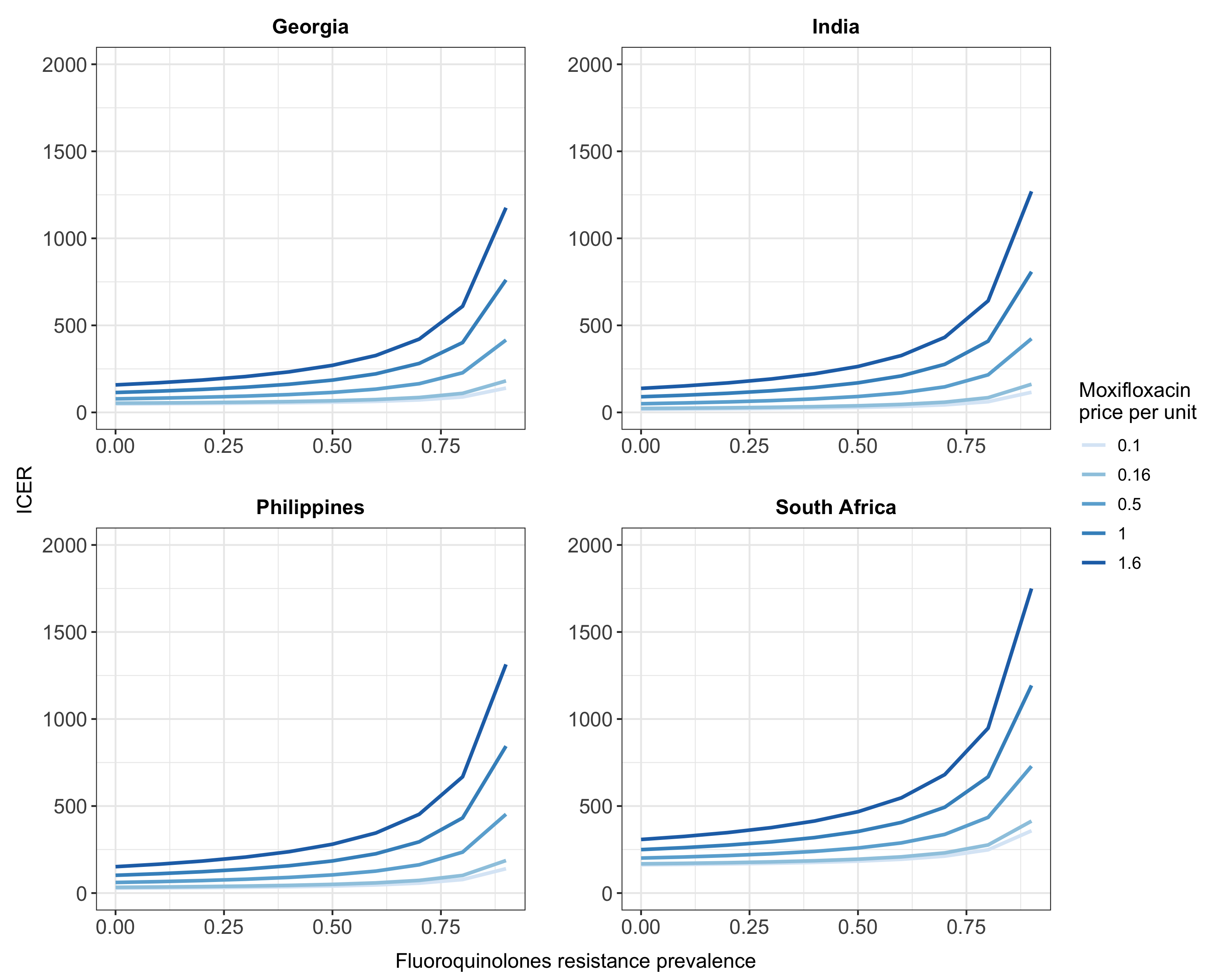

Supplement: S2 Fig — ICER Incremental cost-effectiveness ratio; BPaL bedaquiline, pretomanid and linezolid; BPaLM bedaquiline, pretomanid, linezolid and moxifloxacin; ΔRR, difference in risk ratios. (TIF) [file pgph.0005275.s009.tif]

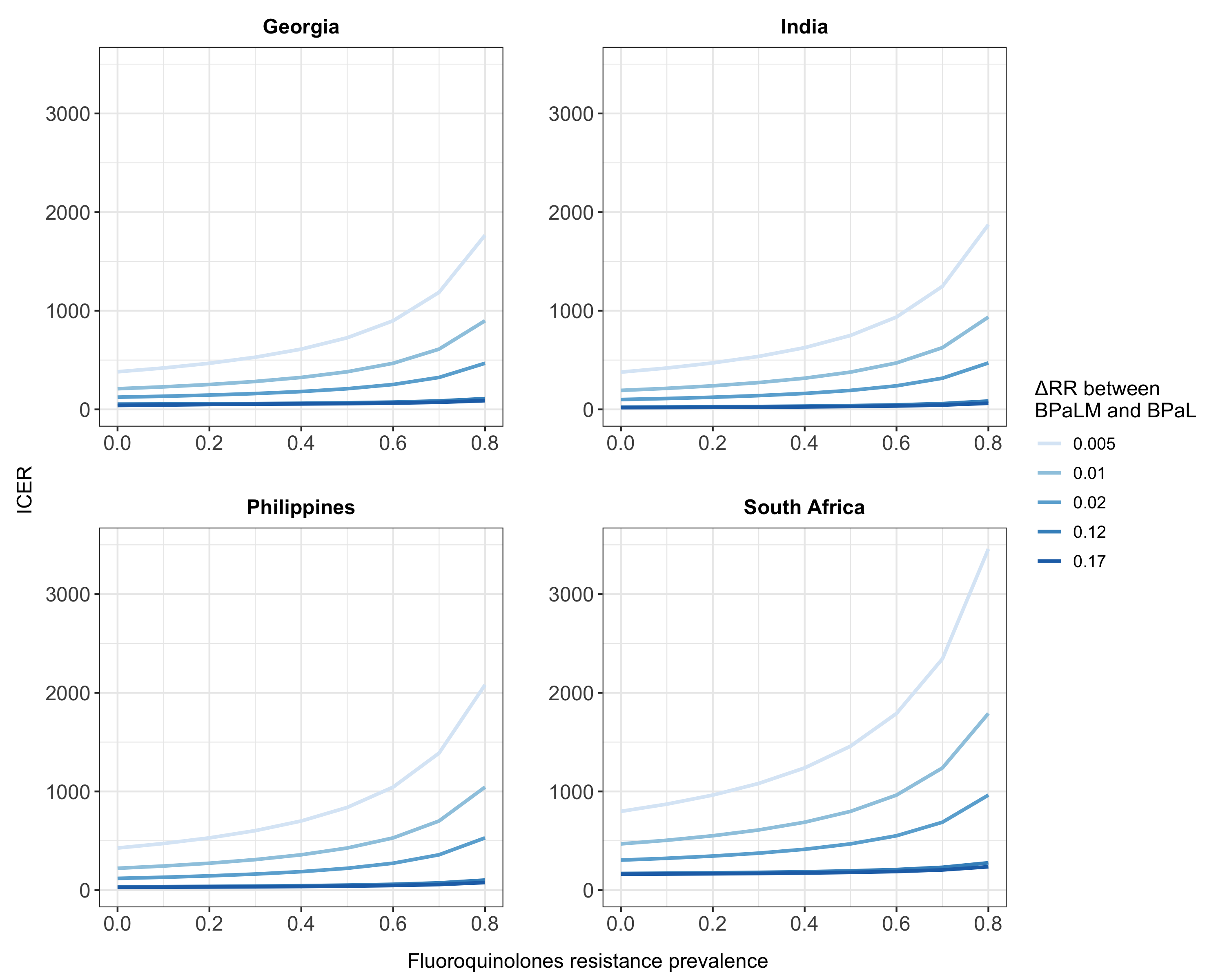

Supplement: S3 Fig — ICER Incremental cost-effectiveness ratio; BPaL bedaquiline, pretomanid and linezolid; BPaLM bedaquiline, pretomanid, linezolid and moxifloxacin; ΔRR, difference in risk ratios. (TIF) [file pgph.0005275.s010.tif]

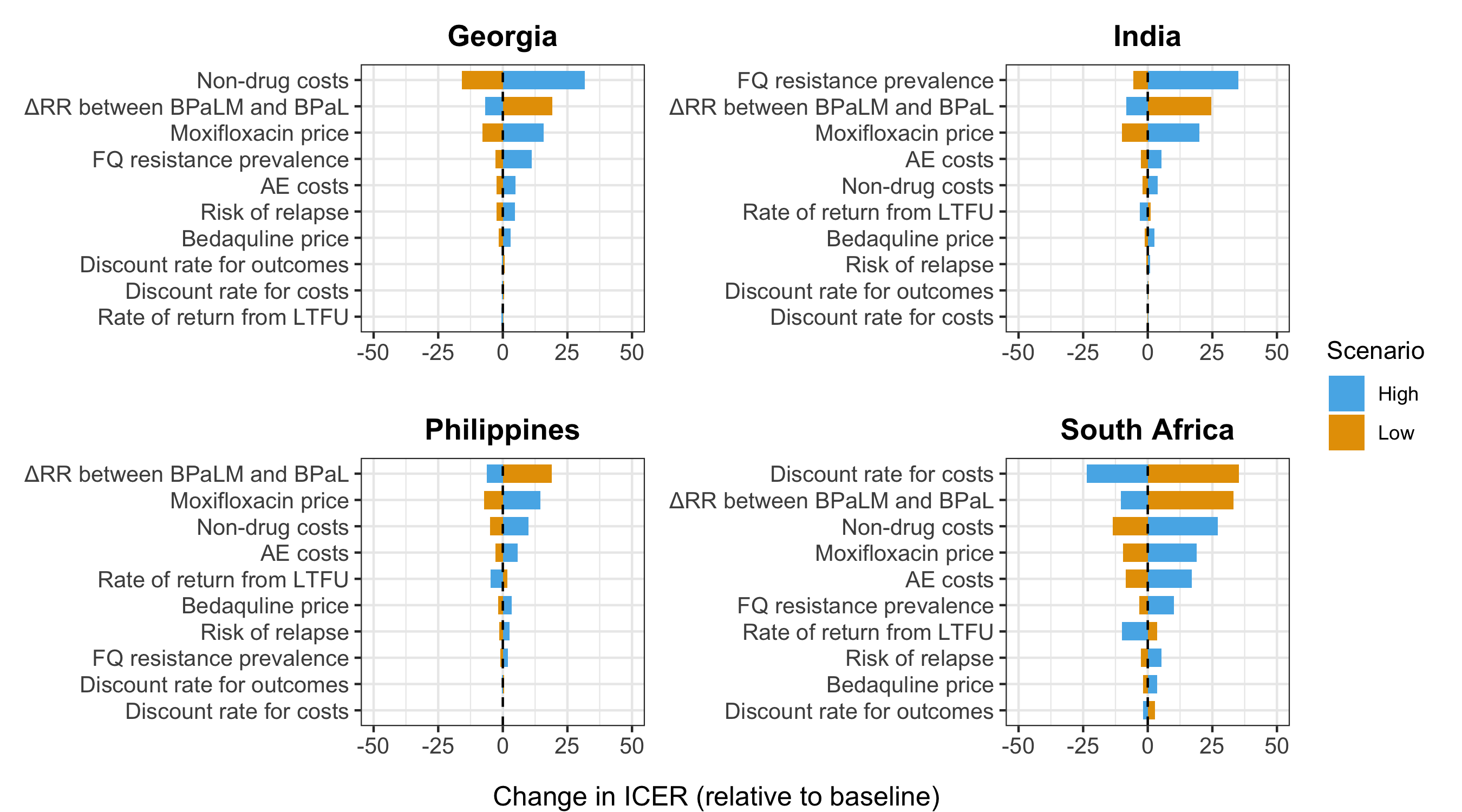

Supplement: S4 Fig — Bars represent the absolute change in ICER (relative to baseline) for high and low values of parameters with all other parameters at baseline values. AE, adverse event; BPaL, bedaquiline, pretomanid and linezolid; BPaLM, bedaquiline, pretomanid, linezolid and moxifloxacin; FQ, fluoroquinolone; ICER, Incremental cost-effectiveness ratio; LTFU, lost to follow-up; ΔRR, difference in risk ratios. (TIF) [file pgph.0005275.s011.tif]

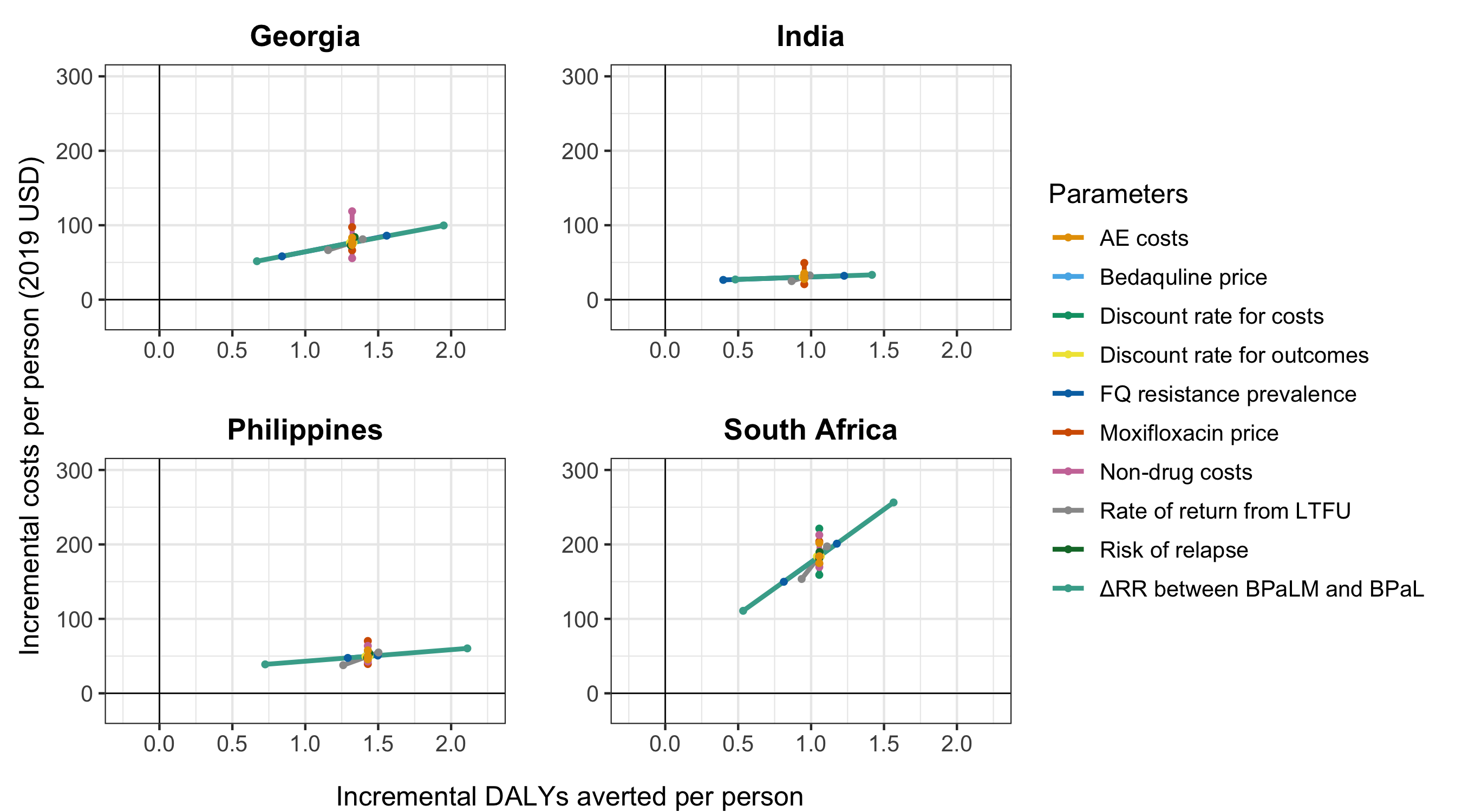

Supplement: S5 Fig — Each line represents the impact of varying a single parameter with all other parameters at baseline values. AE, adverse event; BPaL, bedaquiline, pretomanid and linezolid; BPaLM, bedaquiline, pretomanid, linezolid and moxifloxacin; FQ, fluoroquinolone; ICER, Incremental cost-effectiveness ratio; LTFU, lost to follow-up; ΔRR, difference in risk ratios. (TIF) [file pgph.0005275.s012.tif]
