# Supplementary material for: Impact of fluoroquinolone resistance on the cost-effectiveness of empiric treatment for multidrug- or rifampicin-resistant tuberculosis
Source: PLOS Glob Public Health. 2025 Oct 16;5(10):e0005275. doi: 10.1371/journal.pgph.0005275 (PMC12530546; doi:10.1371/journal.pgph.0005275)
Supplement: Attachment — Submitted filename: Response to Reviewers.pdf [file pgph.0005275.s014.pdf]

Response to Reviewer Comments

Reviewer #1

The document is well written, but my initial impression is that it is more an extension of previous work than new results of public interest. I would suggest that the authors review the introduction to this paper to avoid this perception. Nevertheless, the question of whether it is necessary to introduce a new drug into the treatment scheme for TB should be discussed and studied further.

- Thank you for your valuable feedback. We have revised the introduction to better emphasise the unique contribution of our study. Here is the updated version:
    - (line 77) "Without evidence comparing these regimens under empirical treatment conditions, clinicians must make treatment decisions based on incomplete information… Despite the clinical importance of these treatment decisions, there is currently limited evidence to guide empirical treatment choices between the new all-oral regimens… To address this critical knowledge gap, we conducted the first economic evaluation directly comparing empirical BPaLM versus BPaL treatment across varying fluoroquinolone resistance scenarios. While previous cost-effectiveness analyses assumed DST availability or compared new regimens only against traditional treatments (12,17–19), our study directly compares these two novel short all-oral regimens under different fluoroquinolone resistance scenarios. This analysis addresses a critical evidence gap in empirical treatment settings where DST results are unavailable, providing essential guidance for clinical decision-making."

It is essential to follow the CHEERS statement to clearly state the study perspective, materials and methods, among other things.

- Thank you for this feedback. We have completed and included the CHEERS 2022 checklist as supplementary material (S1 Checklist) and added the following statement to the Methods section.
    - (line 111) The analysis followed the Consolidated Health Economic Evaluation Reporting Standards (CHEERS) 2022, as described in the supplementary materials (**S1 Checklist**) (Husereau et al. 2022).

Given the uncertainty surrounding the parameters used in this exercise, sensitivity analysis should be expanded accordingly.

- Thank you for this feedback. The S2-E Table in our supplementary materials provides detailed information on the uncertainty distributions, standard errors, and parameter ranges used in our sensitivity analyses. We have updated our methods section to provide clearer information as now specified in the revised text:

    - (line 144) To appropriately incorporate the uncertainty in our estimates, we conducted a probabilistic sensitivity analysis (PSA) by varying input parameters such as treatment success rates, unit costs, DALY weights, and transition probabilities over 200 simulation runs, with parameter values, standard errors, and probability distributions specified in **S2-E Table**.
- Also, our sensitivity analysis framework includes univariate analyses across wide parameter ranges, multi-parameter interaction analyses, and cost-effectiveness acceptability curves under varying resistance scenarios, as detailed in the methods section and supplementary materials.

As health economics studies tend to rely more on hypothetical than real data, this should be discussed more in the paper.

- Thank you for this important comment. This is particularly relevant given the limited real-world evidence on treatment success stratified by resistance status, which is why we relied on modelled calculations using back-calculated risk ratios. We have now expanded our discussion of this limitation in the manuscript as follows:
    - (line 254) Our analysis relies on modelled estimates and extrapolated data due to limited real-world evidence on BPaLM effectiveness in routine programmatic settings. Our treatment success estimates were derived from back-calculated risk ratios rather than directly observed country-specific data, where resistance status is often unknown, introducing uncertainty around real-world effectiveness based on resistance status.

In addition, the differences between the four countries' health systems should be further elaborated on to give the reader a better understanding of the results and recommendations.

- Thank you for this important comment. We have added the following elaboration in the discussion better to explain the differences across the four countries:
    - (line 241) The varying incremental cost-effectiveness ratios across countries reflect several interconnected factors, including underlying healthcare cost components, fluoroquinolone resistance prevalence, and health system infrastructure. Countries with higher healthcare service costs, such as South Africa and Georgia, showed correspondingly higher ICERs. Differences in care delivery models, such as the requirement in Georgia for inpatient treatment periods compared to primarily outpatient care elsewhere, may have also contributed to these cost variations. In addition, baseline treatment success rates varied across settings, affecting the potential for improvement with new regimens. Despite these diverse epidemiological, clinical, and health system contexts, the consistent cost-effectiveness of BPaLM as a replacement for BPaL suggests that our findings may be generalisable to similar settings.

Reviewer #2

The authors addressed a significant and timely issue. They estimated the cost-effectiveness of the BPaLM regimen with BPal in the absence of DST for fluoroquinolone. The methods and techniques they utilized are in line with the standards in the literature.

- Thank you for your positive feedback and for recognising the relevance and methodological soundness of our work. We are pleased that our methods and techniques align with the standards in the literature, and we appreciate your thoughtful review.

I have one major and several minor comments.
They indicated one disadvantage of BPaLM over BPAL as more adverse events (Line 60). However, they have not used this information in their analysis. (At least they do not explicitly explain how they used it) Presumably, more adverse events would increase the costs or impact health outcomes or both thus estimated cost effectiveness. Ideally, they could explicitly incorporate these into the decision framework. Since they model the empirical treatment, the decision analytical framework could include the option for physicians to switch the BPaL regime in case of a significant adverse event. If that is not possible due to data availability, some discussion of the issue is warranted in the discussion and limitations sections.

- We thank the reviewer for this important observation regarding the incorporation of adverse events in our analysis. We used (1) adverse event incidence rates from the TB-PRACTECAL trial by regimen, (2) DALY weights from the Global Burden of Disease 2019 study, with expert opinion used to map adverse event symptoms to equivalent GBD health states where not directly specified, and (3) adverse event costs combining unit costs from the Value TB dataset and literature with expert opinion estimates of healthcare service quantities per adverse event. We have clarified this in the method section as follows:
    - (line 153) For the remaining data on costs, DALY weights, and transition probabilities, we used country-specific and non-specific parameters drawn from multiple sources, including national MDR treatment guidelines, the Value TB dataset, published literature, and expert opinion (**S2-E Table**). We incorporated adverse events using the same approach, with aggregate costs derived from TB-PRACTECAL trial incidence data, Global Burden of Disease weights for health impacts, literature-based cost estimates, and expert opinion.
- In our model, patients who failed their initial treatment regimen (BPaL or BPaLM) were switched to a long SOC 'rescue' regimen regardless of the initial treatment they received. However, we did not explicitly model adverse event-related regimen changes separately, given limited data on switching patterns and associated outcomes in empirical treatment settings. This limitation is now acknowledged in the discussion section as follows:
    - (line 274) In addition, all patients who failed their initial treatment regimen were switched to a long SOC 'rescue' regimen, regardless of the initial treatment received. However, we did not explicitly model adverse event-related regimen changes separately due to limited data on switching patterns in empirical settings. This simplified approach may not fully capture the complexity of real-world treatments.

Minor Comments:
• They used 50% country's GDP as the threshold for willingness to pay. Why don't they use the country-specific estimates published in the literature (Woods et al (2016)1, or Riviere et al (2023)2 …)

They used 2019 GDP values; it would have been better if they used more recent GDP levels.

- Thank you for your valuable comments. We have updated the cost-effectiveness thresholds using the estimates provided by Pichon-Riviere et al. (2023). Both Woods et al. (2016) and Pichon-Riviere et al. (2023) are commonly used for cost-effectiveness thresholds in LMICs, but Pichon-Riviere et al. (2023) is the more suitable reference. The Pichon-Riviere paper builds on and updates the earlier work by Woods and Ochalek, making it the most current and comprehensive framework available. Furthermore, the thresholds are reported in 2019 US dollars, which aligns with our cost estimates that are based on 2019 GDP values. Accordingly, we have revised the relevant section of the manuscript as follows:
    - (line 187) Cost-effectiveness acceptability curves (**Figure 2**) indicate that BPaLM has a high probability of being cost-effective compared to BPaL across varying levels of fluoroquinolone resistance. In most scenarios, the probability of cost-effectiveness exceeds 80% even before the willingness-to-pay reaches the country-specific cost-effectiveness thresholds (Georgia $2,419; India $422; the Philippines $962; South Africa $3,951 per DALY averted in USD) (Pichon-Riviere et al. 2023).
- We used 2019 GDP values to build upon the established methodology from Sweeney et al. (2022), which this analysis extends. Using the 2019 GDP also avoids the significant economic distortions caused by the COVID-19 pandemic in subsequent years. In addition, our updated cost-effectiveness thresholds follow the Pichon-Riviere et al. (2023) framework, which provides country-specific thresholds based on 2019 data.

• In Line 70, prevalence rates of fluoroquinolone-resistant TB are given. It is not clear what exactly these rates are. (Among TB patients, among DR-TB, etc?)

- We thank the reviewer for highlighting the need for clarification regarding the prevalence rates of fluoroquinolone-resistant TB mentioned in Line 70. These rates represent the prevalence of fluoroquinolone resistance among MDR/RR-TB cases as defined by the WHO Global TB Report (2024). Specifically, in 2023, 19% (95% CI: 17–21%) of MDR/RR-TB cases globally were classified as pre-XDR-TB, which is defined as resistance to any fluoroquinolone. We have revised the text in the manuscript as follows:
    - (line 72) However, pre-extensively drug-resistant TB (pre-XDR-TB), defined as MDR/RR-TB with additional resistance to fluoroquinolones, was found in 19% of MDR/RR-TB cases globally in 2023, with high setting-specific variance (1).

• In line 128, the authors indicated that "We assumed that the efficacy of BPaLM in the pre-XDR-TB population would be the same as that of BPaL". What is the advantage of this assumption? If I understand equations 1 and 2, that is not necessary.

- Thank you for this insightful question. The assumption that the efficacy of BPaLM in the pre-XDR-TB population is the same as that of BPaL is necessary to solve the equations using the available data. Without this assumption, we would have three unknown parameters (the efficacy of BPaLM in MDR/RR-TB, the efficacy of BPaLM in pre-XDR-TB, and the efficacy of BPaL in both populations), but only two composite efficacy values derived from the PRACTECAL trial results. This would make the equations unsolvable. By assuming equivalence between the efficacy of BPaLM and BPaL in pre-XDR-TB, we reduce the number of unknowns to two, enabling us to back-calculate the population-specific risk ratios. We have also updated the text in the manuscript as follows:
  - (line 129) By assuming that BPaLM efficacy in the pre-XDR-TB population is equivalent to that of BPaL, given the limited role of moxifloxacin in treating TB in fluoroquinolone-resistant strains, we were able to derive the true population-specific risk ratios linked to the regimens (RR_{BPaLM} and RR_{BPaL}):

• In Table 1, RR_BPaLM is smaller than RR_BPaL. Is this a typo? If not, I do not understand that.

- Thank you for this great observation. This was indeed a typo, and it has been corrected as shown in the revised table below.

Parameter	Baseline value	Standard Error	PSA Distribution	Reference
Updated parameters				
BPaL compared to traditional regimen (RR_BPaL)	1.03*	0.05	Log normal	Estimated based on (11) and (13) [Equation 1, 2]
BPaLM compared to traditional regimen (RR_BPaLM)	1.15*	0.06	Log normal	Estimated based on (11) and (13) [Equation 1, 2]

• In Figure 2, including the willingness to pay threshold would make it easier for the audience to comprehend the cost-effectiveness.

- Thank you for this valuable suggestion. Instead of modifying the figure itself, we have included the country-specific thresholds in the figure caption for easy reference as below. This allows readers to easily interpret the results in the context of relevant thresholds for each country.
  - (line 453) Country-specific WTP thresholds are as follows: $2,419 for Georgia, $422 for India, $962 for the Philippines, and $3,951 for South Africa (all in USD) (Pichon-Riviere et al. 2023).

• The authors might explain more elaborately the additional value of their study over the reference 12 and probably reference 18. In order to do that, some discussion about the reasons for the empirical treatment cost of DST for fluoroquinolone would be useful.

- Thank you for your valuable suggestion. We have updated the discussion explaining how our study offers additional value beyond previous analyses as follows:
    - (line 222) "Our analysis addresses a critical gap in the literature by directly comparing the cost-effectiveness of BPaLM versus BPaL regimens in empirical treatment settings across varying fluoroquinolone resistance prevalences… A previous analysis found that BPaL was the most cost-saving option, while BPaLM was preferred at a willingness-to-pay threshold of 0.5 × GDP per disability-adjusted life year averted (12). More recently, a separate analysis using data collected directly from the TB PRACTECAL trial demonstrated the cost-saving potential from a societal perspective of BPaL-based regimens in Belarus, Uzbekistan and South Africa (21). While the available evidence consistently suggests that BPaL-based regimens offer advantages over traditional regimens, these studies did not address the critical question of which regimen to choose in empirical treatment settings. In addition, James et al. (18) compared BPaLM with traditional regimens and examined options for switching regimens if a patient had to discontinue moxifloxacin due to side effects or resistance acquisition. While this analysis considered treatment modifications, it did not directly compare BPaLM with BPaL as initial empirical treatment options, nor did it evaluate cost-effectiveness across varying fluoroquinolone resistance prevalences. Our study addresses this specific gap by providing the first economic comparison of these two WHO-recommended regimens under empirical treatment conditions, accounting for the trade-offs when DST results are unavailable and treatment decisions must be made based on population-level resistance patterns."

• The references to the links in the PDF file go to a lean library account. This makes it difficult to read for non-account holders.

- Thank you for pointing this out. The links in the PDF file are automatically generated by the reference management engine we used during the manuscript preparation. These links will be removed in the final manuscript version.

1. Woods B, Revill P, Sculpher M, Claxton K. Country-Level Cost-Effectiveness Thresholds: Initial Estimates and the Need for Further Research. Value Health. 2016;19(8):929-935. doi:10.1016/j.jval.2016.02.017
2. Pichon-Riviere A, Drummond M, Palacios A, Garcia-Marti S, Augustovski F. Determining the efficiency path to universal health coverage: cost-effectiveness thresholds for 174 countries based on growth in life expectancy and health expenditures. Lancet Glob Health. 2023;11(6):e833-e842. doi:10.1016/S2214-109X(23)00162-6

Reviewer #3

Overview
Thank you for the opportunity to review this manuscript which evaluates cost-effectiveness of BPaLM) compared to BPaL for the empiric treatment of MDR/RR-TB. This is an important study that evaluates realistic scenarios and has potential to directly inform policy and practice in the context of limited or absence of FQ DST.

- Response: We thank Reviewer #3 for the positive assessment of our manuscript and recognition that our study addresses realistic scenarios with direct policy implications.

Comments

What is the potential impact of empiric treatment on wider resistance to fluoroquinolones? Even if this was not evaluated, perhaps it's important to discuss that considering these drugs are used for other conditions and if widespread resistance develops, this would reduce therapeutic options in limited resource settings.

- Response: Thank you for your insightful comments regarding the potential broader impacts of empiric treatment with fluoroquinolones on antimicrobial resistance. We have addressed this point in the discussion section of our manuscript as follows:
    - (line 265) Third, an important consideration not assessed in our analysis is the potential impact of widespread empiric BPaLM use on the development of broader fluoroquinolone resistance. Since fluoroquinolones are used to treat various conditions beyond tuberculosis, their widespread empiric use could contribute to resistance without proper stewardship measures, thereby compromising treatment options for other infections (Goldstein and Garabedian-Ruffalo 2002; Vaughn et al. 2019).

Was this analysis prespecified in a HEAP? This is increasingly being considered good practice.

- Thank you for this point. A formal Health Economic Analysis Plan (HEAP) was not developed prospectively for this analysis. We have completed and included the CHEERS 2022 checklist as supplementary material (S1 Checklist) and added the following statement to the Methods section.
    - (line 111) The analysis followed the Consolidated Health Economic Evaluation Reporting Standards (CHEERS) 2022, as described in the supplementary materials (**S1 Checklist**) (Husereau et al. 2022).

How do 0.5X per capita GDP thresholds compare with opportunity-cost based thresholds published over the past few years? While I think this choice is pragmatic, the absolute values seem large and I wondered if you are able to comment on how the results would be affected by a lower CET of less than 500 USD which is commonly suggested in most LMIC with high TB burden.

- We fully agree that the use of 0.5× GDP per capita can sometimes result in values that exceed opportunity-cost-based estimates, particularly in LMICs with high TB burden. In response to earlier comments, we have updated our analysis using the country-specific cost-effectiveness

thresholds derived by Pichon-Riviere et al. (2023), which are based on health opportunity costs and reflect each country's health system capacity and resource constraints. While we acknowledge that the Pichon-Riviere thresholds are higher than the suggested $500 threshold, these thresholds are country-specific and account for differences in health system capacity, economic development, and resource availability across our study settings. A uniform $500 threshold may not adequately reflect the substantial differences between countries like India and South Africa. Accordingly, we have revised the relevant section of the manuscript as follows:

- (line 187) Cost-effectiveness acceptability curves (**Figure 2**) indicate that BPaLM has a high probability of being cost-effective compared to BPaL across varying levels of fluoroquinolone resistance. In most scenarios, the probability of cost-effectiveness exceeds 80% even before the willingness-to-pay reaches the country-specific cost-effectiveness thresholds (Georgia $2,419; India $422; the Philippines $962; South Africa $3,951 per DALY averted in USD) (Pichon-Riviere et al. 2023).

Journal Requirements:

1. Please ensure you have included the registration number for the clinical trial referenced in the manuscript.

- Thank you for your comment. We have updated the manuscript to include the clinical trial registration numbers for all referenced clinical trials. The updated references are as follows:

8. Esmail A, Oelofse S, Lombard C, Perumal R, Mbuthini L, Goolam Mahomed A, et al. An All-Oral 6-Month Regimen for Multidrug-Resistant Tuberculosis: A Multicenter, Randomized Controlled Clinical Trial (the NExT Study). Am J Respir Crit Care Med. 2022 May 15;205(10):1214-1227. (NCT 02454205).
9. Berry C, du Cros P, Fielding K, Gajewski S, Kazounis E, McHugh TD, et al. TB-PRACTECAL: study protocol for a randomised, controlled, open-label, phase II-III trial to evaluate the safety and efficacy of regimens containing bedaquiline and pretomanid for the treatment of adult patients with pulmonary multidrug-resistant tuberculosis. Trials. 2022 Jun 13;23(1):484. (NCT02589782).
11. Nyang'wa B-T, Berry C, Kazounis E, Motta I, Parpieva N, Tigay Z, et al. Short oral regimens for pulmonary rifampicin-resistant tuberculosis (TB-PRACTECAL): an open-label, randomised, controlled, phase 2B-3, multi-arm, multicentre, non-inferiority trial. Lancet Respir Med. 2024 Feb;12(2):117-128. (NCT02589782).

2. Please amend your detailed Financial Disclosure statement. This is published with the article. It must therefore be completed in full sentences and contain the exact wording you wish to be published.

Please clarify all sources of funding (financial or material support) for your study. List the grants (with grant number) or organizations (with url) that supported your study, including funding received from your institution.

State the initials, alongside each funding source, of each author to receive each grant.

State what role the funders took in the study. If the funders had no role in your study, please state: "The funders had no role in study design, data collection and analysis, decision to publish, or preparation of the manuscript."
If any authors received a salary from any of your funders, please state which authors and which funders.

If you did not receive any funding for this study, please simply state: "The authors received no specific funding for this work."

- Thanks for this comment. We have updated the Financial Disclosure statement as follows:
  - (line 295) This study was funded by the UK Medical Research Council London Intercollegiate Doctoral Training Partnership Studentship (grant number MR/W006677/1) awarded to Chaelin Kim. The UK Medical Research Council London

Intercollegiate Doctoral Training Partnership is a partnership between City, St George's University of London, and London School of Hygiene & Tropical Medicine (https://mrc-lid.lshtm.ac.uk/). The funder had no role in study design, data collection and analysis, decision to publish, or preparation of the manuscript. The authors received no other specific funding for this work.

3. Please provide separate figure files in .tif or .eps format.

For more information about figure files please see our guidelines:
https://journals.plos.org/globalpublichealth/s/figures
https://journals.plos.org/globalpublichealth/s/figures#loc-file-requirements

- We have attached the figure files in the updated .eps format as per the PLOS Global Public Health guidelines.

4. Your manuscript is missing the following sections: Abstract. Please ensure these are present, and in the correct order, and that any references to subheadings in your main text are correct. An outline of the required sections can be consulted in our submission guidelines here:
https://journals.plos.org/globalpublichealth/s/submission-guidelines#loc-parts-of-a-submission

- We have updated the abstract section of the manuscript according to the PLOS Global Public Health submission guidelines.

5. We have noticed that you have uploaded Supporting Information files, but you have not included a list of legends. Please add a full list of legends for your Supporting Information files after the references list.

- We have added the full list of Supporting Information legends after the references in the updated main manuscript.

6. If the reviewer comments include a recommendation to cite specific previously published works, please review and evaluate these publications to determine whether they are relevant and should be cited. There is no requirement to cite these works unless the editor has indicated otherwise.

- Thank you for your comment. Based on the reviewer's recommendations, we have carefully reviewed and evaluated the suggested publications for their relevance to our manuscript. We have included the following new reference that is directly relevant to our analysis:

  21. Pichon-Riviere A, Drummond M, Palacios A, Garcia-Marti S, Augustovski F. Determining the efficiency path to universal health coverage: cost-effectiveness thresholds for 174 countries based on growth in life expectancy and health expenditures. Lancet Glob Health. 2023 Jun;11(6):e833–42.

7. Please review your reference list to ensure that it is complete and correct. If you have cited papers that have been retracted, please include the rationale for doing so in the manuscript text, or remove these references and replace them with relevant current references. Any changes to the reference list

should be mentioned in the rebuttal letter that accompanies your revised manuscript. If you need to cite a retracted article, indicate the article's retracted status in the References list and also include a citation and full reference for the retraction notice.

- Thank you for your comment. We have thoroughly reviewed our reference list to ensure it is complete and correct. All updates to the reference list have been highlighted in yellow in the revised manuscript.
